# The integrated comprehension of lncRNA HOXA-AS3 implication on human diseases

**DOI:** 10.1007/s12094-022-02920-w

**Published:** 2022-08-20

**Authors:** Qinfan Yao, Cuili Wang, Yucheng Wang, Xiuyuan Zhang, Hong Jiang, Dajin Chen

**Affiliations:** 1 Kidney Disease Center, The First Affiliated Hospital, College of Medicine, Zhejiang University, Qingchun Road 79, Hangzhou, 310003 China; 2 Key Laboratory of Kidney Disease Prevention and Control Technology, Hangzhou, Zhejiang Province China; 3 National Key Clinical Department of Kidney Diseases, Institute of Nephrology, Zhejiang University, Hangzhou, China; 4 Zhejiang Clinical Research Center of Kidney and Urinary System Disease, Hangzhou, China

**Keywords:** HOXA-AS3, Long non-coding RNAs, Tumor promoter, Mechanism, Clinical applications

## Abstract

Long non-coding RNA (lncRNA) is a non-protein-coding RNA with a length of more than 200 nucleotides. Studies have shown that lncRNAs have vital impacts on various pathological processes and participate in the development of human diseases, usually through acting as competing endogenous RNAs to modulate miRNA expression and biological functions. lncRNA HOXA Cluster Antisense RNA 3 (HOXA-AS3) was a newly discovered lncRNA and has been demonstrated to be abnormally expressed in many diseases. Moreover, HOXA-AS3 expression was closely correlated with the clinicopathologic characteristics in cancer patients. In addition, HOXA-AS3 exhibited significant properties in regulating several biological processes, including cell proliferation, invasion, and migration. Furthermore, HOXA-AS3 has provided promising values in the diagnosis, prognosis, and therapeutic strategies of several diseases such as liver cancer, glioma, lung cancer, oral cancer, gastric cancer, and even atherosclerosis. In this review, we discuss the abnormal expression of HOXA-AS3 in several human disorders and some pathobiological processes and its clinical characteristics, followed by a summary of HOXA-AS3 functions, regulatory mechanisms, and clinical application potential.

## Introduction

Long non-coding RNA (lncRNA) represents a non-coding functional RNA subtype with over 200 nucleotides in length [1–5]. Along with a growing number of long non-coding RNAs (lncRNAs) being identified in recent years, investigating the biological functions of lncRNAs has gained increasing attention [5–9]. Increasing evidence suggests that lncRNA dysregulation is linked to illness onset and progression, particularly malignancies [10–13]. In addition, functional investigations have also indicated that lncRNAs play an essential role in the pathogenesis of various diseases via several molecular processes, including cell proliferation, metabolism, migration, invasion, and apoptosis [12, 14]. Furthermore, lncRNAs have emerged as novel focuses of clinical applications due to the increasing in-depth studies on molecular mechanisms [14–18] for the functions of numerous lncRNAs [17, 19–21].

lncRNA HOXA Cluster Antisense RNA 3 (HOXA-AS3) is a newly discovered lncRNA with 25,952 bases and in the genomic location at human chromosome 7p15.2 started 900 nt downstream of the 3’ end of HOXB5 [22]. HOXA-AS3 expression has been implicated in a variety of human diseases and pathophysiological processes, including liver cancer [23–25], glioma [26, 27], lung cancer [28–30], oral cancer [31], colorectal cancer [32], gastric cancer [33], pancreatic cancer [34], endometriosis [35], atherosclerosis [36, 37], pulmonary arterial hypertension [38, 39], and even the lineage differentiation of mesenchymal stem cells (MSCs) [39], according to numerous studies. In these disorders, high HOXA-AS3 expression has been reported to closely relate to several clinicopathologic characteristics, such as pathological grade, TNM stage, tumor size, lymph node metastasis, invasion depth, and *Helicobacter pylori* infection status, overall survival, and disease-free survival. Research has further revealed that HOXA-AS3 exerted regulatory effects on the initiation and progression of various human disease types through the positive induction of many cellular processes such as cell proliferation, apoptosis, migration, invasion, chemotherapy resistance, endothelium inflammation, and MSCs differentiation. Furthermore, HOXA-AS3 has been shown to have a high potential for a variety of interesting therapeutic applications in diagnosis, prognosis, and therapy. In this review, we focus on the expression profiles, corresponding clinicopathologic features, biological roles, molecular mechanisms, and clinical applications of HOXA-AS3 in diverse disease types and pathophysiological processes.

### The expression of HOXA-AS3 and its roles in human diseases and pathophysiological processes

Recent evidence showed that the overexpression of HOXA-AS3 was revealed in various types of human diseases and pathophysiological processes, including liver cancer [23–25], glioma [26, 27], lung cancer [28–30], oral cancer [31], colorectal cancer [32], gastric cancer [33], pancreatic cancer [34], endometriosis [35], atherosclerosis [36, 37], pulmonary arterial hypertension [38], and the lineage differentiation of mesenchymal stem cells (MSCs) [39]. In addition, high HOXA-AS3 expression has been identified to correlate with unfavorable clinicopathological features and poor prognoses, such as pathological grade, TNM stage, tumor size, lymph node metastasis, invasion depth, and *Helicobacter pylori* infection status, overall survival, and disease-free survival (Table 1). HOXA-AS3 was also involved in regulating biological functions and disease processes through various mechanisms, including cell proliferation, apoptosis, migration, invasion, drug resistance, endothelium inflammation, and MSCs lineage specification (Table 2). This section briefly introduces HOXA-AS3 expression changes, relevant clinicopathologic features, and the leading biological roles in diverse disease types and pathophysiological processes.

**Table 1:** lncRNA HOXA-AS3 expression and clinical characteristics in human diseases and pathophysiological processes

Disease type	Expression	Clinical characteristics	Refs
Liver cancer	Overexpression	Overall survival	[23–25]
Glioma	Overexpression	Overall survival and pathological grade	[26, 27]
Lung cancer	Overexpression	–	[28–30]
Oral cancer	Overexpression	Pathological stage, and overall survival	[31]
Colorectal cancer	Overexpression	–	[32]
Gastric cancer	Overexpression	Tumor size, lymph node status, invasion depth, *Helicobacter pylori* infection status, over survival, and disease-free survival	[33]
Pancreatic cancer	Overexpression	Poor prognosis, TNM stage, and lymph node metastasis	[34]
Atherosclerosis	Overexpression	Pathological conditions of the coronary wall, the levels of TG, TC, and LDL-C	[36, 37]
Pulmonary arterial hypertension	Overexpression	–	[38]
MSCs lineage determination	Overexpression	–	[39]

**Table 2:** Roles and regulatory mechanisms of lncRNA HOXA-AS3 in human diseases and pathophysiological processes

Disease type	Role	Cell lines	Functions	Related mechanisms	Refs
Liver cancer	Tumor promoter	Hep3B, SNU-387, Li-7, SMMC-7721, HepG2, Huh7, and HCC-LM3	Cell proliferation, apoptosis, migration, and invasion	miR-29c, BMP1, miR-455-5p, and PD-L1	[23–25]
Glioma	Tumor promoter	LN229, U251, SNB19, U87, U138, and H4	Cell proliferation, apoptosis, and migration	miR-455-5p, and USP3	[25, 27]
Lung cancer	Tumor promoter	A549, PC-9, NCI-H358, and NCI-H1299	Cell proliferation, migration, invasion, cisplatin resistance	HOXA3, NF110, and HOXA6	[28–30]
Oral cancer	Tumor promoter	TSCCA, CAL-27, SCC-9, and Tca8113	Cell proliferation	miR-218-5p	[31]
Colorectal cancer	Tumor promoter	SW480, SW620, HCT116, COLO205, and LOVO	Cell proliferation, and apoptosis	miR-4319, SPNS2, and AKT	[32]
Gastric cancer	Tumor promoter	MGC-803, AGS, MKN45, SGC7901, and HGC-27	Cell proliferation, migration, and invasion	miR-29a-3p, and LTβR	[33]
Pancreatic cancer	Tumor promoter	Panc-1, Aspc-1, sw1990, and Bxpc-3	Cell proliferation	miR-29c, and CDK6	[34]
Atherosclerosis	–	HUVECs	Endothelium inflammation, cell proliferation, and apoptosis	NF-κB, miR-455-5p, p27, and Kip1	[34, 37]
Pulmonary arterial hypertension	–	HPASMCs	Cell proliferation, apoptosis, migration, and invasion	miR-675-3p, and PDE5A	[38]
MSCs lineage determination	–	MSCs	Osteogenic differentiation	EZH2, and RUNX2	[39]

## Cancers

### Liver cancer

It was found that HOXA-AS3 expression was markedly upregulated in hepatocellular carcinoma cells (Hep3B, SNU-387, Li-7, SMMC-7721, HepG2, Huh7, and HCC-LM3) and tissues [23–25]. In addition, patients with high HOXA-AS3 levels were confirmed to possess shorter overall survival. Similarly, functional studies revealed that HOXA-AS3 increased cell proliferation, anti-apoptosis, migration, invasion, the epithelial–mesenchymal transition (EMT), and the MEK/ERK signaling pathway in Hep3B, HuH-7, SMMC-7721, and HepG2 cells, as well as tumor growth and lung metastasis in mouse xenograft models [23–25].

### Glioma

HOXA-AS3 was highly expressed in glioma tissues and cell lines (LN229, U251, SNB19, U87, U138, and H4) and was closely associated with poor prognoses such as worse overall survival and pathological grade [26, 27]. HOXA-AS3 was demonstrated, through functional experiments, to facilitate the processes of cell proliferation, anti-apoptosis, and migration in LN229, H4, and U251 cells and in vivo tumor xenograft models [26, 27].

### Lung cancer

In small cell lung cancer, HOXA-AS3 expression was shown to upregulate over fourfold in patients’ tissues acquired stable disease (SD)/progressive disease (PD) after first-line chemotherapy compared to partial response (PR) patients’ tissues [28]. Furthermore, in a dose- and time-dependent manner, HOXA-AS3 was dramatically overexpressed in non-small-cell lung cancer tissues and cell lines after cisplatin therapy [29, 30]. Moreover, HOXA-AS3 exerted chemoresistance functions by inducing anti-apoptosis and EMT in A549, PC-9, NCI-H358, and NCI-H1299 cells. The developed xenograft mice model confirmed that HOXA-AS3 knockdown increased cisplatin effectiveness in lung cancer [30].

### Oral cancer

In addition, HOXA-AS3 was also highly expressed in oral squamous cell carcinoma tissues and cell lines (TSCCA, CAL-27, SCC-9, and Tca8113) [31]. Furthermore, high levels of HOXA-AS3 indicated an undesirable pathological stage and overall survival. Moreover, HOXA-AS3 has also played proliferative roles on SCC-9 and CAL-27 cells [31].

### Colorectal cancer

Overexpression of HOXA-AS3 has been reported in colorectal cancer tissues and SW480, SW620, HCT116, COLO205, and LOVO cells. In addition, HOXA-AS3 showed strong abilities to stimulate cell proliferation, suppress cell apoptosis in COLO205 and LOVO cell lines, and accelerate tumor growth in vivo [32].

### Gastric cancer

HOXA-AS3 was upregulated in gastric cancer cell lines (MGC-803, AGS, MKN45, SGC7901, and HGC-27) and tissues. In addition, high HOXA-AS3 levels were correlated with poor prognosis, including tumor size, lymph node status, invasion depth, *Helicobacter pylori* infection status, over survival, and disease-free survival. More crucially, HOXA-AS3 increased cell proliferation, migration, and invasion in MKN45 and SGC7901 cells and tumor development and lung metastasis in vivo [33].

### Pancreatic cancer

HOXA-AS3 was overexpressed in pancreatic cancer tissues and Panc-1, Aspc-1, sw1990, and Bxpc-3 cells and was closely bound up with worse prognosis, aggressive TNM stage, and lymph node metastasis. HOXA-AS3 was also reported to exist in its pro-proliferative effects for Panc-1 and Bxpc-3 cells as well as subcutaneous xenograft tumors [34].

## Non-cancerous diseases

### Atherosclerosis

The elevation of HOXA-AS3 was observed in human umbilical vein endothelial cells (HUVECs) and associated with worse pathological conditions of the coronary wall, increased levels of TG, TC and LDL-C in the serum of mice model. By promoting HUVEC adherence to monocytes and monocyte movement across HUVEC monolayers [36], HOXA-AS3 was thought to be a crucial activator for endothelium inflammation. HOXA-AS3 was also involved in the anti-proliferative and apoptotic effects of ox-LDL-induced HUVECs and the advancement of angiogenesis [37].

### Pulmonary arterial hypertension

HOXA-AS3 was also overexpressed hypoxia-treated human pulmonary artery smooth muscle cells (HPASMCs), which was regarded as in vitro model of pulmonary arterial hypertension (PAH). Besides, HOXA-AS3 has been proved to promote proliferation and migration but repress the apoptosis of HPASMCs [38].

### Mesenchymal stem cells lineage commitment

The expression of HOXA-AS3 in mesenchymal stem cells (MSCs) was increased during adipogenic differentiation, whereas it was unchanged during osteogenic differentiation. Moreover, HOXA-AS3 has been proposed as a critical factor in epigenetic tuning that contributed to the lineage differentiation of MSCs. Downregulation of HOXA-AS3 in both human MSCs and mouse MSCs resulted in improved osteogenesis and impaired adipogenesis [39].

### The regulatory mechanisms of HOXA-AS3 in human diseases and pathophysiological processes

Numerous studies have found that lncRNAs primarily work by interacting with miRNAs and interfering with gene expression [40–44]. Functional analysis indicated that HOXA-AS3 participated in regulating various cell biological processes via numerous mechanisms, including cell proliferation, apoptosis, invasion, metastasis, chemotherapy sensitivity, endothelium inflammation, and MSCs-lineage differentiation. Following that, we will discuss the molecular mechanisms of fundamental biological tasks such as cell proliferation, migration, and invasion. Uncontrolled cell proliferation constitutes malignant transformation and eventually tumor occurrence [44–48]. Meanwhile, cell migration and invasion are indispensable properties for cancer metastasis, mainly attributed to cancer-related death [49–53].

**Fig. 1:**
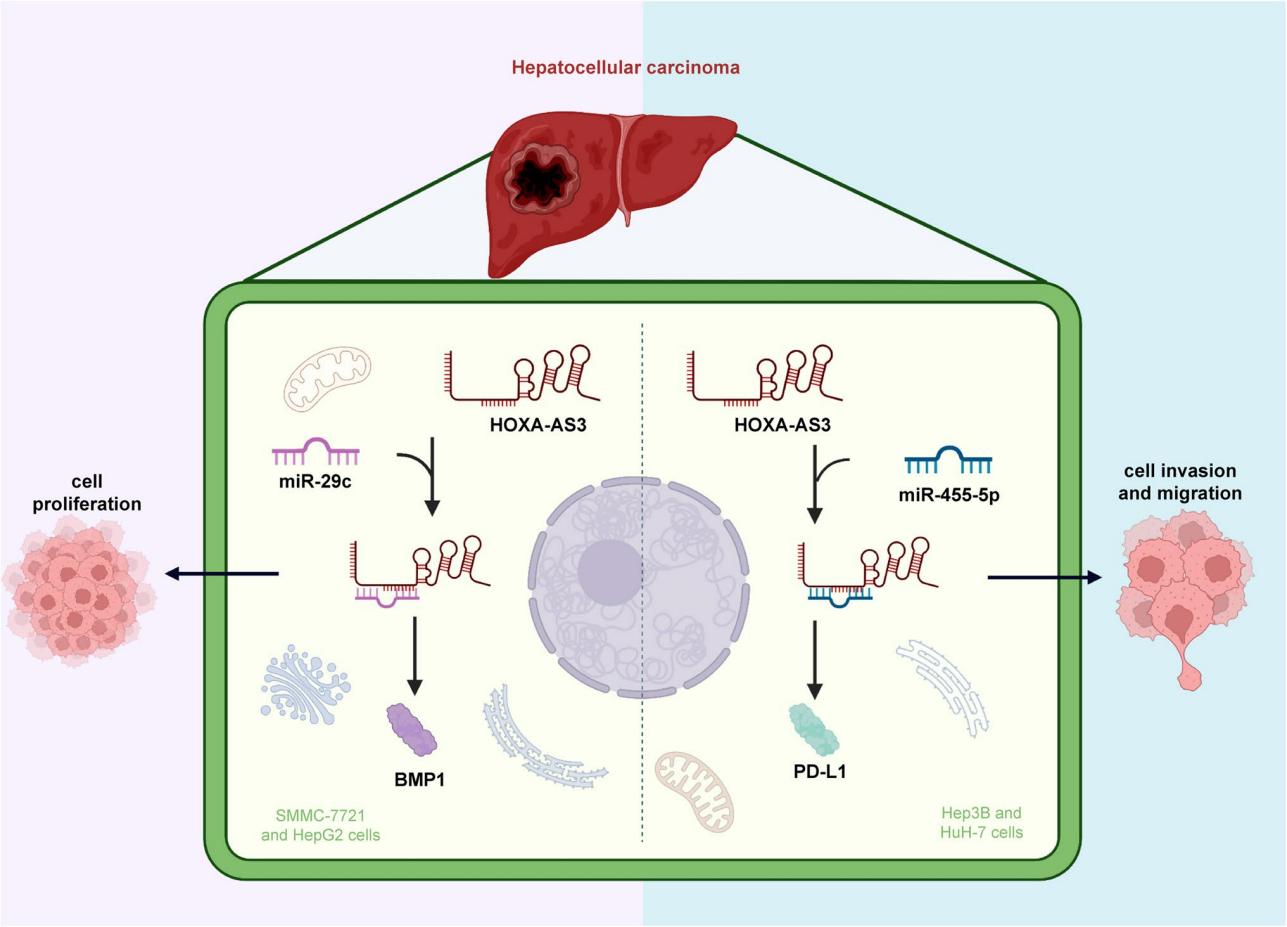
In hepatocellular carcinoma, HOXA-AS3 exerted its functions in regulating cell proliferation, invasion, and migration by interacting with miR-29c to upregulate BMP1 expression or combining with miR-455-5p to increase PD-L1 level

It is clear that HOXA-AS3 exerts several functions in many cancer types. In hepatocellular carcinoma, HOXA-AS3 enhanced cell proliferation, migration, invasion, and the epithelial–mesenchymal transition (EMT) process by interacting with miR-29c to increase BMP1 expression in SMMC-7721 and HepG2 cells, as well as binding to miR-455-5p to upregulate PD-L1 expression in Hep3B and HuH-7 cells (Fig. 1) [23, 24]. Moreover, HOXA-AS3 was also shown to sponge miR-455-5p to increase USP3 levels in glioma LN229 and H4 cells, allowing them to proliferate, and migrate [27]. In addition, HOXA-AS3 combined with HOXA3 to induce EMT and inhibit cell apoptosis in non-small-cell lung carcinoma cells (A549, PC-9, NCI-H358, and NCI-H1299), therefore, weakening the effectiveness of cisplatin treatment [29]. Furthermore, in lung adenocarcinoma A549 cells, HOXA-AS3 was shown to be triggered by histone acylation and then bound to NF110 to raise HOXA6 levels, leading to cell proliferation, migration, and invasion [30]. In oral squamous cell carcinoma SCC-9 and CAL-27 cells, HOXA-AS3 performed the pro-proliferative functions through the interaction with miR-218-5p [31]. HOXA-AS3 also strengthened colorectal cancer cell proliferation and apoptosis in COLO205 and LOVO cells by repressing the expression of miR-4319 to activate SPNS2 expression and AKT signaling pathway [32]. In gastric cancer MKN45 and SGC7901 cells, HOXA-AS3 has been found to accelerate the processes of cell proliferation, migration, and invasion via restraining miR-29a-3p from raising the LTβR expression and then sensitizing the NF-κB signaling pathway [33]. Moreover, HOXA-AS3 exerts a pro-proliferative action in pancreatic cancer Panc-1 and Bxpc-3 cells through the activation of NF-κB signaling.

Recently, HOXA-AS3 was also shown to play pivotal roles in non-cancer diseases. During the process of inflammatory atherosclerosis, HOXA-AS3 was revealed to enhance monocyte migration through HUVEC monolayers through the activation of NF-κB signaling and exhibit an anti-proliferative and apoptotic effect on ox-LDL induced HUVECs via miR-455-5p/p27 Kip1 axis. In osteogenic differentiation of MSCs, HOXA-AS3 regulated the lineage specification of MSC by combining with EZH2 and also influencing the H3 lysine-27 trimethylation of Runx2 [39]. Besides, HOXA-AS3 combined with miR-675-3p to elevate PDE5A levels, boosting the proliferation, migration, and anti-apoptosis of hypoxia-treated human pulmonary artery smooth muscle cells [38].

### The potential application of HOXA-AS3 in clinical practice

#### HOXA-AS3 as a promising diagnostic and prognostic marker

lncRNAs are considered potential biomarkers in illness diagnosis and prognosis based on the properties of tissue-specific lncRNA expression patterns and their extensive participation in numerous biological processes [54–57]. Recently, multiple published studies have shown the significance of HOXA-AS3 in the diagnostic and prognostic potentials for diverse diseases. The differences in the expression of HOXA-AS3 in a variety of diseases can be used to distinguish diseased from adjacent normal tissues and cell lines, contributing to good diagnostic value for disease screening. Moreover, the strong correlation between HOXA-AS3 expression with the clinical features in various disease types has also been confirmed to possess solid prognostic value for disease risk assessment. For example, Kaplan–Meier analysis demonstrated that patients with high HOXA-AS3 expression suffered lower overall survival rates and even disease-free survival rates. HOXA-AS3 expression was also verified as an independent prognostic predictor in various disorders, including hepatocellular carcinoma, glioma [26, 27], oral squamous cell carcinoma, and gastric cancer, using univariate and multivariate Cox regression analyses [23–25]. Moreover, the expression profile of HOXA-AS3 increased in a dose- and time-dependent manner after cisplatin treatment, suggesting the potential of HOXA-AS3 to predict cisplatin resistance [29]. Nevertheless, HOXA-AS3 expression was currently detected in only tissues and cell lines, while have not been extensively performed on blood and other accessible body fluids. In addition, restricted accessibility of tumor tissue, inconvenient tissue storage, traumatic nature, and high cost make tissue biopsy an unsatisfactory choice for clinical applications [58–64]. Based on the several deficiencies of biopsy tissues, non-invasive and minimally invasive body fluid assessments are more conducive to early disease identification, more convenient for monitoring disease prognosis repeatedly, and more helpful in guiding timely intervention and treatment [65–69]. Consequently, it is necessary to explore further the specificity and stability of HOXA-AS3 expression in biological samples with less invasive (such as blood and urine) in different diseases.

#### HOXA-AS3 as a prospective treatment target

lncRNAs are involved in various malignant biological behaviors, and understanding the potential role of lncRNAs in diseases represents a new perspective to develop molecular targeted therapeutic strategies [70–74]. Previously, it has been reported that HOXA-AS3 was overexpressed in numerous disorders and exerted a critical role in driving the disease formation and progression. Besides, numerous studies have further shown that HOXA-AS3 modulated the biological processes of disease types through several mechanisms. HOXA-AS3 have been demonstrated to function as competing endogenous RNAs (ceRNA) by sponging miRNA and inhibiting miRNA function. Targeting HOXA-AS3 to repress its abnormal upregulation has been shown to suppress disease progression in many disease models, representing a potent approach to developing therapeutic agents for several types of disease. For example, suppression of HOXA-AS3 in glioma tumor models showed an apparent reduction in glioma tumor weight and size, suggesting the promising therapeutic potential of targeted-HOXA‐AS3 for glioma [27]. Besides, downregulation of HOXA-AS3 by CRISPR-dCas9 has also been indicated to impair the tumor growth of pancreatic cancer [34] Panc-1 cells in vivo. Moreover, the 5′-terminal region of HOXA-AS3 from nt 1 to 800 was verified to be associated with the modulation of NF-κB activity and, therefore, targeting the region of HOXA-AS3 from nt 1 to 800 may act as a promising therapeutic strategy for the treatment of multiple NF-κB-mediated inflammatory disorders [36]. Besides, HOXA-AS3 knockdown also dramatically alleviated the symptom of atherosclerosis and ameliorated the pathological change in the coronary wall, the levels of TG, TC, and LDL-C in serum of mice, exhibiting the powerful therapeutic effect on atherosclerosis [37].

In addition, chemoresistance [75–78] remains a primary obstacle in cancer therapy. Elucidating the underlying mechanism of chemoresistance could improve the curative effect of chemotherapy and guide the effective therapeutic method [79–83]. It has been reported that HOXA-AS3 expression was implicated in the development of small cell lung cancer chemotherapy [28] insensitivity and non-small-cell lung carcinoma cisplatin [29] resistance, knockdown of HOXA-AS3 enhanced the efficacy of chemotherapeutic drugs in lung cancer.

These results suggest that suppressing HOXA‐AS3 expression may have therapeutic potential for numerous diseases. Moreover, it may have significant implications for studying and treating different clinical disorders. However, the clinical treatment strategies based on HOXA‐AS3-targeted agents have not yet been broadly applied. In future research, the safety, stability, and efficacy of HOXA‐AS3-targeted drugs should be adequately evaluated in large-scale randomized clinical trials. It is a promising field of HOXA‐AS3 in non-invasive disease detection, prognosis, and target treatment.

## Conclusion

HOXA-AS3 expression was upregulated in several human disorders and pathophysiological processes, such as liver cancer, glioma, lung cancer, oral cancer, colorectal cancer, gastric cancer, pancreatic cancer, endometriosis, atherosclerosis, pulmonary arterial hypertension, and the osteogenic differentiation of MSCs. Furthermore, HOXA-AS3 overexpression was tightly associated with numerous clinicopathological features, such as tumor size, pathological grade, lymph node metastasis, infection status, overall survival, and disease-free survival. In addition, HOXA-AS3 also played a crucial role in regulating biological functions and participated in the pathogenesis of diseases by multiple mechanisms. It is also well established that HOXA-AS3 was considered a promising biomarker and had a beneficial impact on clinical applications, including diagnosis, prognosis, and treatment. Additional in vivo and clinical experiments of identifying HOXA-AS3 expression characteristics in non-invasive samples and testing the efficacy and safety of targeted-HOXA-AS3 drugs will provide helpful insights into future disease management.

## Data Availability

Not applicable.

## References

[1] Zhang XW, Li QH, Xu ZD, Dou JJ (2021). STAT1-induced regulation of lncRNA ZFPM2-AS1 predicts poor prognosis and contributes to hepatocellular carcinoma progression via the miR-653/GOLM1 axis. Cell Death Dis.

[2] Schmitz SU, Grote P, Herrmann BG (2016). Mechanisms of long noncoding RNA function in development and disease. Cell Mol Life Sci.

[3] Wu R, Li L, Bai Y, Yu B, Xie C, Wu H (2020). The long noncoding RNA LUCAT1 promotes colorectal cancer cell proliferation by antagonizing Nucleolin to regulate MYC expression. Cell Death Dis.

[4] Liu X, She Y, Wu H, Zhong D, Zhang J (2018). Long non-coding RNA Gas5 regulates proliferation and apoptosis in HCS-2/8 cells and growth plate chondrocytes by controlling FGF1 expression via miR-21 regulation. J Biomed Sci.

[5] Barth DA, Juracek J, Slaby O, Pichler M, Calin GA (2020). lncRNA and mechanisms of drug resistance in cancers of the genitourinary system. Cancers (Basel).

[6] Cao MX, Jiang YP, Tang YL, Liang XH (2017). The crosstalk between lncRNA and microRNA in cancer metastasis: orchestrating the epithelial-mesenchymal plasticity. Oncotarget.

[7] Yuan JH, Yang F, Wang F, Ma JZ, Guo YJ, Tao QF (2014). A long noncoding RNA activated by TGF-β promotes the invasion-metastasis cascade in hepatocellular carcinoma. Cancer Cell.

[8] Lanzafame M, Bianco G, Terracciano LM, Ng CKY, Piscuoglio S (2018). The role of long non-coding RNAs in hepatocarcinogenesis. Int J Mol Sci.

[9] Zhang Y, Pitchiaya S, Cieślik M, Niknafs YS, Tien JC, Hosono Y (2018). Analysis of the androgen receptor-regulated lncRNA landscape identifies a role for ARLNC1 in prostate cancer progression. Nat Genet.

[10] Galasso M, Dama P, Previati M, Sandhu S, Palatini J, Coppola V (2014). A large scale expression study associates uc 283-plus lncRNA with pluripotent stem cells and human glioma. Genome Med..

[11] Xue Z, Hennelly S, Doyle B, Gulati AA, Novikova IV, Sanbonmatsu KY (2016). A G-rich motif in the lncRNA braveheart interacts with a zinc-finger transcription factor to specify the cardiovascular lineage. Mol Cell.

[12] Yu JE, Ju JA, Musacchio N, Mathias TJ, Vitolo MI (2020). Long noncoding RNA DANCR activates Wnt/β-catenin signaling through MiR-216a inhibition in non-small cell lung cancer. Biomolecules.

[13] Liu CY, Zhang YH, Li RB, Zhou LY, An T, Zhang RC (2018). LncRNA CAIF inhibits autophagy and attenuates myocardial infarction by blocking p53-mediated myocardin transcription. Nat Commun.

[14] Xiao ZD, Han L, Lee H, Zhuang L, Zhang Y, Baddour J (2017). Energy stress-induced lncRNA FILNC1 represses c-Myc-mediated energy metabolism and inhibits renal tumor development. Nat Commun.

[15] Chen Y, Li X, Li B, Wang H, Li M, Huang S (2019). Long non-coding RNA ECRAR triggers post-natal myocardial regeneration by activating ERK1/2 signaling. Mol Ther.

[16] Lai L, Li H, Feng Q, Shen J, Ran Z (2021). Multi-factor mediated functional modules identify novel classification of ulcerative colitis and functional gene panel. Sci Rep.

[17] Mosca L, Vitiello F, Borzacchiello L, Coppola A, Tranchese RV, Pagano M (2021). Mutual correlation between non-coding RNA and S-adenosylmethionine in human cancer: roles and therapeutic opportunities. Cancers (Basel).

[18] Cai H, Yu Y, Ni X, Li C, Hu Y, Wang J (2020). LncRNA LINC00998 inhibits the malignant glioma phenotype via the CBX3-mediated c-Met/Akt/mTOR axis. Cell Death Dis.

[19] Yuan L, Xu ZY, Ruan SM, Mo S, Qin JJ, Cheng XD (2020). Long non-coding RNAs towards precision medicine in gastric cancer: early diagnosis, treatment, and drug resistance. Mol Cancer.

[20] Geng W, Lv Z, Fan J, Xu J, Mao K, Yin Z (2021). Identification of the prognostic significance of somatic mutation-derived LncRNA signatures of genomic instability in lung adenocarcinoma. Front Cell Dev Biol.

[21] Jiang R, Zhang H, Zhou J, Wang J, Xu Y, Zhang H (2021). Inhibition of long non-coding RNA XIST upregulates microRNA-149-3p to repress ovarian cancer cell progression. Cell Death Dis.

[22] Degani N, Lubelsky Y, Perry RB, Ainbinder E, Ulitsky I (2021). Highly conserved and cis-acting lncRNAs produced from paralogous regions in the center of HOXA and HOXB clusters in the endoderm lineage. PLoS Genet.

[23] Tong Y, Wang M, Dai Y, Bao D, Zhang J, Pan H (2019). LncRNA HOXA-AS3 sponges miR-29c to facilitate cell proliferation, metastasis, and EMT process and activate the MEK/ERK signaling pathway in hepatocellular carcinoma. Hum Gene Ther Clin Dev.

[24] Zeng C, Ye S, Chen Y, Zhang Q, Luo Y, Gai L (2021). HOXA-AS3 promotes proliferation and migration of hepatocellular carcinoma cells via the miR-455-5p/PD-L1 axis. J Immunol Res.

[25] Ye J, Wu S, Pan S, Huang J, Ge L (2020). Risk scoring based on expression of long non-coding RNAs can effectively predict survival in hepatocellular carcinoma patients with or without fibrosis. Oncol Rep.

[26] Wu F, Zhang C, Cai J, Yang F, Liang T, Yan X (2017). Upregulation of long noncoding RNA HOXA-AS3 promotes tumor progression and predicts poor prognosis in glioma. Oncotarget.

[27] Chen W, Li Q, Zhang G, Wang H, Zhu Z, Chen L (2020). LncRNA HOXA-AS3 promotes the malignancy of glioblastoma through regulating miR-455-5p/USP3 axis. J Cell Mol Med.

[28] Kuang P, Chen P, Wang L, Li W, Chen B, Liu Y (2020). RNA sequencing analysis of small cell lung cancer reveals candidate chemotherapy insensitivity long noncoding RNAs and microRNAs. Ann Transl Med.

[29] Lin S, Zhang R, An X, Li Z, Fang C, Pan B (2019). LncRNA HOXA-AS3 confers cisplatin resistance by interacting with HOXA3 in non-small-cell lung carcinoma cells. Oncogenesis.

[30] Zhang H, Liu Y, Yan L, Zhang M, Yu X, Du W (2018). Increased levels of the long noncoding RNA, HOXA-AS3, promote proliferation of A549 cells. Cell Death Dis.

[31] Zhao Y, Yao R (2021). Long non-coding RNA HOXA-AS3 promotes cell proliferation of oral squamous cell carcinoma through sponging microRNA miR-218-5p. Bioengineered.

[32] Jiang Y, Yu XY, Sun HX, Gu XY, Geng JS (2021). Long non-coding RNA HOXA-AS3 facilitates the malignancy in colorectal cancer by miR-4319/SPNS2 axis. J Physiol Biochem.

[33] Qu F, Zhu B, Hu YL, Mao QS, Feng Y (2021). LncRNA HOXA-AS3 promotes gastric cancer progression by regulating miR-29a-3p/LTβR and activating NF-κB signaling. Cancer Cell Int.

[34] Zhang X, Zhu H, Qu X, Yu Z, Zhang J (2021). Suppressing LncRNA HOXA-AS3 by CRISPR-dCas9 inhibits pancreatic cancer development. J Cancer.

[35] Gu C, Meng Y, Meng Q, Fan W, Ye M, Zhang Q (2021). Exploring the potential key IncRNAs with endometriosis by construction of a ceRNA network. Int J Gen Med.

[36] Zhu X, Chen D, Liu Y, Yu J, Qiao L, Lin S (2019). Long noncoding RNA HOXA-AS3 integrates NF-κB signaling to regulate endothelium inflammation. Mol Cell Biol.

[37] Chi K, Zhang J, Sun H, Liu Y, Li Y, Yuan T (2020). Knockdown of lncRNA HOXA-AS3 suppresses the progression of atherosclerosis via sponging miR-455-5p. Drug Des Devel Ther.

[38] Li ZK, Gao LF, Zhu XA, Xiang DK (2021). LncRNA HOXA-AS3 promotes the progression of pulmonary arterial hypertension through mediation of miR-675-3p/PDE5A axis. Biochem Genet.

[39] Zhu XX, Yan YW, Chen D, Ai CZ, Lu X, Xu SS (2016). Long non-coding RNA HoxA-AS3 interacts with EZH2 to regulate lineage commitment of mesenchymal stem cells. Oncotarget.

[40] Xia Y, Zhen L, Li H, Wang S, Chen S, Wang C (2021). MIRLET7BHG promotes hepatocellular carcinoma progression by activating hepatic stellate cells through exosomal SMO to trigger Hedgehog pathway. Cell Death Dis.

[41] Ma MZ, Li CX, Zhang Y, Weng MZ, Zhang MD, Qin YY (2014). Long non-coding RNA HOTAIR, a c-Myc activated driver of malignancy, negatively regulates miRNA-130a in gallbladder cancer. Mol Cancer.

[42] Parfenyev S, Singh A, Fedorova O, Daks A, Kulshreshtha R, Barlev NA (2021). Interplay between p53 and non-coding RNAs in the regulation of EMT in breast cancer. Cell Death Dis.

[43] Yan H, Liu Q, Jiang J, Shen X, Zhang L, Yuan Z (2021). Identification of sex differentiation-related microRNA and long non-coding RNA in *Takifugu* rubripes gonads. Sci Rep.

[44] Karreth FA, Pandolfi pp.  (2013). ceRNA cross-talk in cancer: when ce-bling rivalries go awry. Cancer Discov.

[45] Dewal N, Freedman ML, LaFramboise T, Pe'er I (2010). Power to detect selective allelic amplification in genome-wide scans of tumor data. Bioinformatics.

[46] Szabó A, Merks RMH (2017). Blood vessel tortuosity selects against evolution of aggressive tumor cells in confined tissue environments: a modeling approach. PLoS Comput Biol.

[47] Hanahan D, Weinberg RA (2011). Hallmarks of cancer: the next generation. Cell.

[48] Ramundo V, Giribaldi G, Aldieri E (2021). Transforming growth factor-β and oxidative stress in cancer: a crosstalk in driving tumor transformation. Cancers (Basel).

[49] Kotini M, Barriga EH, Leslie J, Gentzel M, Rauschenberger V, Schambony A (2018). Gap junction protein Connexin-43 is a direct transcriptional regulator of N-cadherin in vivo. Nat Commun.

[50] Vazquez K, Saraswathibhatla A, Notbohm J (2022). Effect of substrate stiffness on friction in collective cell migration. Sci Rep.

[51] Cheng SY, Chen NF, Lin PY, Su JH, Chen BH, Kuo HM (2019). Anti-invasion and antiangiogenic effects of stellettin B through inhibition of the Akt/Girdin signaling pathway and VEGF in glioblastoma cells. Cancers (Basel).

[52] Mayor R, Etienne-Manneville S (2016). The front and rear of collective cell migration. Nat Rev Mol Cell Biol.

[53] Limia CM, Sauzay C, Urra H, Hetz C, Chevet E, Avril T (2019). Emerging roles of the endoplasmic reticulum associated unfolded protein response in cancer cell migration and invasion. Cancers (Basel).

[54] Sun CC, Zhu W, Li SJ, Hu W, Zhang J, Zhuo Y (2020). FOXC1-mediated LINC00301 facilitates tumor progression and triggers an immune-suppressing microenvironment in non-small cell lung cancer by regulating the HIF1α pathway. Genome Med.

[55] Ding C, Shan Z, Li M, Xia Y, Jin Z (2021). Exploration of the associations of lncRNA expression patterns with tumor mutation burden and prognosis in colon cancer. Onco Targets Ther.

[56] Yu J, Mao W, Sun S, Hu Q, Wang C, Xu Z (2021). Identification of an m6A-Related lncRNA signature for predicting the prognosis in patients with kidney renal clear cell carcinoma. Front Oncol.

[57] Yu G, Lin J, Liu C, Hou K, Liang M, Shi B (2017). Long non-coding RNA SPRY4-IT1 promotes development of hepatic cellular carcinoma by interacting with ERRα and predicts poor prognosis. Sci Rep.

[58] Nagao K, Inada T, Tamura A, Kajitani K, Shimamura K, Yukawa H (2018). Circulating markers of collagen types I, III, and IV in patients with dilated cardiomyopathy: relationships with myocardial collagen expression. ESC Heart Fail.

[59] Wang HW, Peng CY, Lai HC, Su WP, Lin CH, Chuang PH (2017). New noninvasive index for predicting liver fibrosis in Asian patients with chronic viral hepatitis. Sci Rep.

[60] Divgi CR, Uzzo RG, Gatsonis C, Bartz R, Treutner S, Yu JQ (2013). Positron emission tomography/computed tomography identification of clear cell renal cell carcinoma: results from the REDECT trial. J Clin Oncol.

[61] Yu-Wai-Man C, Khaw PT (2016). Personalized medicine in ocular fibrosis: myth or future biomarkers. Adv Wound Care (New Rochelle).

[62] Temilola DO, Wium M, Coulidiati TH, Adeola HA, Carbone GM, Catapano CV (2019). The prospect and challenges to the flow of liquid biopsy in africa. Cells.

[63] Zhao D, Zhou T, Luo Y, Wu C, Xu D, Zhong C (2021). Preliminary clinical experience applying donor-derived cell-free DNA to discern rejection in pediatric liver transplant recipients. Sci Rep.

[64] Luddi A, Zarovni N, Maltinti E, Governini L, Leo V, Cappelli V (2019). Clues to non-invasive implantation window monitoring: isolation and characterisation of endometrial exosomes. Cells.

[65] Mathai RA, Vidya RVS, Reddy BS, Thomas L, Udupa K, Kolesar J (2019). Potential utility of liquid biopsy as a diagnostic and prognostic tool for the assessment of solid tumors: implications in the precision oncology. J Clin Med.

[66] Fernandes MGO, Sousa C, Jacob M, Almeida L, Santos V, Araújo D (2021). Resistance profile of osimertinib in pre-treated patients with EGFR T790M-Mutated non-small cell lung cancer. Front Oncol.

[67] Tatischeff I (2021). Current search through liquid biopsy of effective biomarkers for early cancer diagnosis into the rich cargoes of extracellular vesicles. Int J Mol Sci.

[68] Li L, Hann HW, Wan S, Hann RS, Wang C, Lai Y (2016). Cell-free circulating mitochondrial DNA content and risk of hepatocellular carcinoma in patients with chronic HBV infection. Sci Rep.

[69] Smolle E, Taucher V, Lindenmann J, Pichler M, Smolle-Juettner FM (2021). Liquid biopsy in non-small cell lung cancer-current status and future outlook-a narrative review. Transl Lung Cancer Res.

[70] Wang X, Guo Y, Wang C, Wang Q, Yan G (2021). Long noncoding RNA ZEB1-AS1 downregulates miR-23a, promotes tumor progression, and predicts the survival of oral squamous cell carcinoma patients. Onco Targets Ther.

[71] Zhu J, Chen S, Yang B, Mao W, Yang X, Cai J (2019). Biosci Rep.

[72] Dodd DW, Gagnon KT, Corey DR (2013). Digital quantitation of potential therapeutic target RNAs. Nucleic Acid Ther.

[73] Jiang L, Li H, Fan Z, Zhao R, Xia Z (2019). Circular RNA expression profiles in neonatal rats following hypoxic-ischemic brain damage. Int J Mol Med.

[74] Chen F, Qi S, Zhang X, Wu J, Yang X, Wang R (2019). lncRNA PLAC2 activated by H3K27 acetylation promotes cell proliferation and invasion via the activation of Wnt/β-catenin pathway in oral squamous cell carcinoma. Int J Oncol.

[75] Zhou Y, Wang K, Zhou Y, Li T, Yang M, Wang R (2020). HEATR1 deficiency promotes pancreatic cancer proliferation and gemcitabine resistance by up-regulating Nrf2 signaling. Redox Biol.

[76] Levin M, Stark M, Berman B, Assaraf YG (2019). Surmounting Cytarabine-resistance in acute myeloblastic leukemia cells and specimens with a synergistic combination of hydroxyurea and azidothymidine. Cell Death Dis.

[77] Vahedi S, Lusvarghi S, Pluchino K, Shafrir Y, Durell SR, Gottesman MM (2018). Mapping discontinuous epitopes for MRK-16, UIC2 and 4E3 antibodies to extracellular loops 1 and 4 of human P-glycoprotein. Sci Rep.

[78] Bahar E, Kim JY, Yoon H (2019). Chemotherapy resistance explained through endoplasmic reticulum stress-dependent signaling. Cancers (Basel).

[79] Ye Y, Yang S, Han Y, Sun J, Xv L, Wu L (2019). HOXD-AS1 confers cisplatin resistance in gastric cancer through epigenetically silencing PDCD4 via recruiting EZH2. Open Biol.

[80] Sui X, Chen R, Wang Z, Huang Z, Kong N, Zhang M (2013). Autophagy and chemotherapy resistance: a promising therapeutic target for cancer treatment. Cell Death Dis.

[81] Moon JY, Manh Hung LV, Unno T, Cho SK (2018). Nobiletin enhances chemosensitivity to adriamycin through modulation of the Akt/GSK3β/β− Catenin/MYCN/MRP1 signaling pathway in A549 human non-small-cell lung cancer cells. Nutrients.

[82] Xu J, Shi Q, Xu W, Zhou Q, Shi R, Ma Y (2019). Metabolic enzyme PDK3 forms a positive feedback loop with transcription factor HSF1 to drive chemoresistance. Theranostics.

[83] Xie L, Shi F, Li Y, Li W, Yu X, Zhao L (2020). Drp1-dependent remodeling of mitochondrial morphology triggered by EBV-LMP1 increases cisplatin resistance. Signal Transduct Target Ther.

